# A Hybrid NRPS-PKS Gene Cluster Related to the Bleomycin Family of Antitumor Antibiotics in *Alteromonas* *macleodii* Strains

**DOI:** 10.1371/journal.pone.0076021

**Published:** 2013-09-19

**Authors:** Carolina Megumi Mizuno, Nikole E. Kimes, Mario López-Pérez, Eva Ausó, Francisco Rodriguez-Valera, Rohit Ghai

**Affiliations:** Evolutionary Genomics Group, Departamento de Producción Vegetal y Microbiologia, Universidad Miguel Hernández, San Juan de Alicante, Alicante, Spain; Institut National de la Recherche Agronomique, France

## Abstract

Although numerous marine bacteria are known to produce antibiotics via hybrid NRPS-PKS gene clusters, none have been previously described in an *Alteromonas* species. In this study, we describe in detail a novel hybrid NRPS-PKS cluster identified in the plasmid of the *Alteromonas* *macleodii* strain AltDE1 and analyze its relatedness to other similar gene clusters in a sequence-based characterization. This is a mobile cluster, flanked by transposase-like genes, that has even been found inserted into the chromosome of some *Alteromonas* *macleodii* strains. The cluster contains separate genes for NRPS and PKS activity. The sole PKS gene appears to carry a novel acyltransferase domain, quite divergent from those currently characterized. The predicted specificities of the adenylation domains of the NRPS genes suggest that the final compound has a backbone very similar to bleomycin related compounds. However, the lack of genes involved in sugar biosynthesis indicates that the final product is not a glycopeptide. Even in the absence of these genes, the presence of the cluster appears to confer complete or partial resistance to phleomycin, which may be attributed to a bleomycin-resistance-like protein identified within the cluster. This also suggests that the compound still shares significant structural similarity to bleomycin. Moreover, transcriptomic evidence indicates that the NRPS-PKS cluster is expressed. Such sequence-based approaches will be crucial to fully explore and analyze the diversity and potential of secondary metabolite production, especially from increasingly important sources like marine microbes.

## Background

Marine bacteria are gaining prominence as producers of secondary metabolites, which display unique structural and functional characteristics [1,2]. This emerging source of natural products is of particular importance due to a decline in antibiotic drug discovery from their traditional source (i.e., soil microbes) over the past two decades [3]. Polyketides (PKs) and non-ribosomal peptides (NRPs) represent one of the larger classes of marine microbial natural products with important clinical and ecological impacts [4]. Although different in overall structure and fundamental chemical building blocks, they exhibit striking similarities in their biosynthetic assembly mechanisms [5]. Polyketide synthases (PKS) and non-ribosomal peptide synthetases (NRPS), large modular enzymes, are responsible for the synthesis of PKs and NRPs, respectively. The striking similarities between PKs and NRPSs, both structural and catalytic, allow for the formation of hybrid clusters that contain elements of each class [5]. These hybrid NRPS-PKS clusters provide even greater variety for potential secondary metabolites produced by microorganisms [6].

Hybrid NRPS-PKS compounds have been isolated from numerous marine bacteria, many of them produced by gram-positive actinomycetes [7,8]. Genomic evidence suggests that these hybrid systems are common [9], and secondary metabolite production has been described in many cases. Although much of the focus has been on actinomycetes, other microorganisms, including gram-negative marine bacteria, also contain NRPS-PKS hybrid systems. *Vibrio* spp., for example, produce the antibiotic Andrimide using a hybrid NRPS-PKS system [10]. In addition, *Roseobacter* spp. exhibit the genetic potential for the production of secondary metabolites utilizing similar systems [11].

*Alteromonas* species are known to produce ecologically and clinically relevant natural products. For example, Alteramide A, a tetracyclic alkaloid produced by an *Alteromonas* sp. associated with the sponge *Halichondriaokadai* exhibits cytotoxic and antimicrobial activity [12]. Numerous *Alteromonas* isolates have also exhibit algicidal activities. *Alteromonas* sp. KNS-16, for example, was isolated from a harmful algal bloom in Korea [13] and *Alteromonas* sp. strain A14 has been shown to reduce blooms of *Cochlodiniumpolykrikoides* (a dinoflagellate) [14], leading some to consider the use of *Alteromonas* in containing such blooms [13]. Furthermore, the closely related genus *Pseudoalteromonas* has been shown to harbor hybrid NRPS-PKS systems, as described in *Pseudoalteromonas* sp. strain NJ631 [15] and to produce antimicrobial compounds, such as Thiomarinal [16,17,18].

*Alteromonas* *macleodii* was first isolated off the coast of Hawaii by Baumann [19] and has subsequently been found to be widely distributed throughout the world, including the Mediterranean [20,21,22]. Mesocosm studies have demonstrated that *Alteromonas* was among the most active microorganisms in nutrient enrichment experiments [23], while more recent metatranscriptomic studies have provided further evidence that *A* *. macleodii* is a rapidly-growing microbe upon nutrient availability in otherwise oligotrophic conditions [24], exhibiting typical *r*-strategist behavior. The algicidal activity displayed by *Alteromonas* isolates [13,14] appears perfectly coupled to their *r*-strategist nature, exploiting a brief overabundance of nutrients made available during events like a red-tide and declining rapidly thereafter.

The genomes of two highly related *A* *. macleodii* strains, AltDE and AltDE1, which were isolated from a single water sample collected from the Adriatic Sea (1000m) [25], have been sequenced [25,26]. Their genomic comparison revealed that AltDE1, unlike AltDE, carries a 300 kb plasmid (henceforth pAMDE1). Interestingly, a large gene cluster coding for several non-ribosomal peptide synthetases (NRPS) and a polyketide synthase (PKS) genes is present in pAMDE1 [26]. Moreover, *A* *. macleodii* strains also isolated from the Mediterranean [27], contain this same cluster in either a plasmid, like AltDE1, or inserted in the chromosome, suggesting that it is a mobile cluster [22]. We now describe this novel hybrid NRPS-PKS in *A* *. macleodii* strain AltDE1 and provide evidence that suggests strong parallels between the hybrid NRPS-PKS cluster of *A* *. macleodii* and gene clusters involved in biosynthesis of bleomycin family of antitumor antibiotics produced by actinomycetes [28].

## Methods

### Domain Annotation

NRPS and PKS domains were identified in the cluster using the SBSPKS server [29]. NRPSpredictor2 [30] and the SBSPKS server [29] were used to identify binding specificity of the A domains in the NRPS modules. I-TASSER [31] was used to identify the AT domain associated with the PKS module. All-vs-all comparisons of the cluster sequences were performed using BLAST. Structural alignment of known protein structures of 1MLA, 3QAT, 2JFD and bleomycin family acyltransferase domains was performed using the PROMALS3D web server [32].

### Phylogenetic analysis

A representative set of sequences of the beta-ketoacyl synthase (KS) and the Condensation domains (C) were obtained from the NAPDOS server [33]. Sequences were aligned using MUSCLE [34] and the alignments were trimmed using trimAl [35]. Maximum likelihood trees were constructed using RAxML [36] with the JTT matrix under a gamma model of rate heterogeneity and estimation of the alpha parameter.

### Phleomycin resistance

Each *A* *. macleodii* strain (i.e., AltDE, AltDE1, UM7, U4, U7, and U8) was grown on marine agar (MA; 3.5% sea salt, 0.5% peptone, 0.1% yeast extract, and 1.5% agar). Individual colonies were grown in marine broth (MB; 3.5% sea salt, 0.5% peptone, 0.1% yeast extract) at 25°C overnight. Each culture was adjusted to an OD600 of 1.0, and 100 µL inoculated onto MA plates pretreated with Phleomycin (Invivogen, San Diego, CA, USA). The pretreated MA plates were prepared by adding either 50 or 100 µg of Phleomycin (from a 20mg/mL stock) drop wise to each plate in three different regions. After allowing the antibiotic to dry (c.a., 30 min), the individual strains were plated across an entire plate. Resistance was evaluated after two days of growth at either 25°C or 13°C. A strain was considered susceptible if there was a clear zone (no growth) around the antibiotic spots. Biological duplicates were performed.

### Transcriptomic analysis

Using -80°C glycerol stocks, AltDE1 was grown on marine agar (3.5% sea salt, 0.5% peptone, 0.1% yeast extract, and 1.5% agar) and examined for purity. An individual colony was grown in marine broth (MB, 3.5% sea salt, 0.5% peptone, 0.1% yeast extract) at 25°C overnight, and 4 mL of the inoculum was transferred to 96 mL of MB. The optical density (OD_600_) was measured using a BioPhotometer (Eppendorf), and 150 µL of 1.0 OD600 AltDE and AltDE1 was added to 15 mL of MB. The culture was grown to an OD600 of 0.8 (mid-exponential growth phase) and 2X RNAlater® Solution (Ambion AM7024) was then added. The cultures were centrifuged at 5000 rpm for 10 min, and the RNA was extracted from the cell pellets using RNeasy® (Qiagen 74106). The RNA was treated with DNAse I at room temp for 30 min, deactivated at 65°C for 10 min after adding the stop solution, and purified using ethanol precipitation. Agarose gel electrophoresis and staining confirmed the absence of genomic DNA in the RNA. Total RNA (10 µg) was used to make single-stranded cDNA using High Capacity cDNA Reverse Transcription (Applied Biosystems 4368814) as per the manufacturer’s instructions. The second strand was synthesized by adding 30 U of *E. coli* Polymerase I (New England Biolabs M0209L), 5 U of *E. coli* DNA Ligase (New England Biolabs M0205S), 5 U of RNase H (Epicentre R52250), 300 µM of dNTPs (Invitrogen 18427-013) to the first strand reaction. After 2 h at 16 °C, the double-stranded cDNA was cleaned with a QIAquick PCR Purification kit (Qiagen 28104) and quantified using the ND-1000 Spectrophotometer (NanoDrop, Wilmington, USA). The cDNA was sequenced using the Illumina HiSeq 2000 platform (100-bp paired-end read, GATC Biotech), resulting in ca. 8,000,000 reads that were subsequently mapped to the AltDE1 genome [37], providing over 10X coverage of the genome. Gene expression is presented as the commonly used reads per kilobase per million mapped (RPKM) values which normalize for the transcript length in kilobases [38].

To validate the RNA-sequencing results PCR amplification from AltDE1 cDNA was performed using BIOTAQ DNA polymerase (BioLine BIO-21040) in 50 µL reactions as follows: 37.5 µL sterile water, 5 µL 10X reaction buffer, 4 µL of MgCl2 (25 mg/mL), 1 µL of a dNTP mix (10 mM), 1 µL each primer (10 µM) and 0.5 µL BIOTAQ polymerase (5U/µl). All PCR reactions were performed on a PTC-100 Peltier Thermal Cycler (MJ Research Inc.). Three genes from pAMDE1 were amplified using the following primers: ORF 94, a NRPS gene (94f 5’ ACGGGTTGCAGGGGGTCGTA3’ and 94r 5’ TGTGCGGTGCGAGGCAAAGT3’); pAMDE1-102, the PKS gene ORF 102 (102f 5’ ACGACGGTGCGCTGAACCTG3’ and 102r 5’ AGCCAACGCACACTCGTCCG3’) and ORF 108, a NRPS gene (108f 5’ CGCCACAAAGGCGCAGGAGA3’ and 108r 5’ GCCGCAACGCATTGGCGAAA3’). The temperature cycling profile for the 16S gene amplification was 1 cycle at 95°C for 5 min; 30 cycles at 94°C for 45 s, 57°C for 45 s and 72°C for 2 min; and 1 cycle at 72°C for 10 min. The temperature cycling profiles for the three pAMDE1 genes were 1 cycle at 95°C for 5 min; 35 cycles at 94°C for 30 s, 55°C for 30 s and 72°C for 1 min; and 1 cycle at 72°C for 10 min. All PCR amplification products were visualized using electrophoresis on 0.8% agarose gels with a 100bp ladder as a size reference.

### Accession numbers

Transciptomic sequence data have been deposited in the INSDC Sequence Read Archive under the accession SRP028786.

## Results and Discussion

### General Features of the pAMDE1 hybrid NRPS-PKS cluster

**Figure 1:**
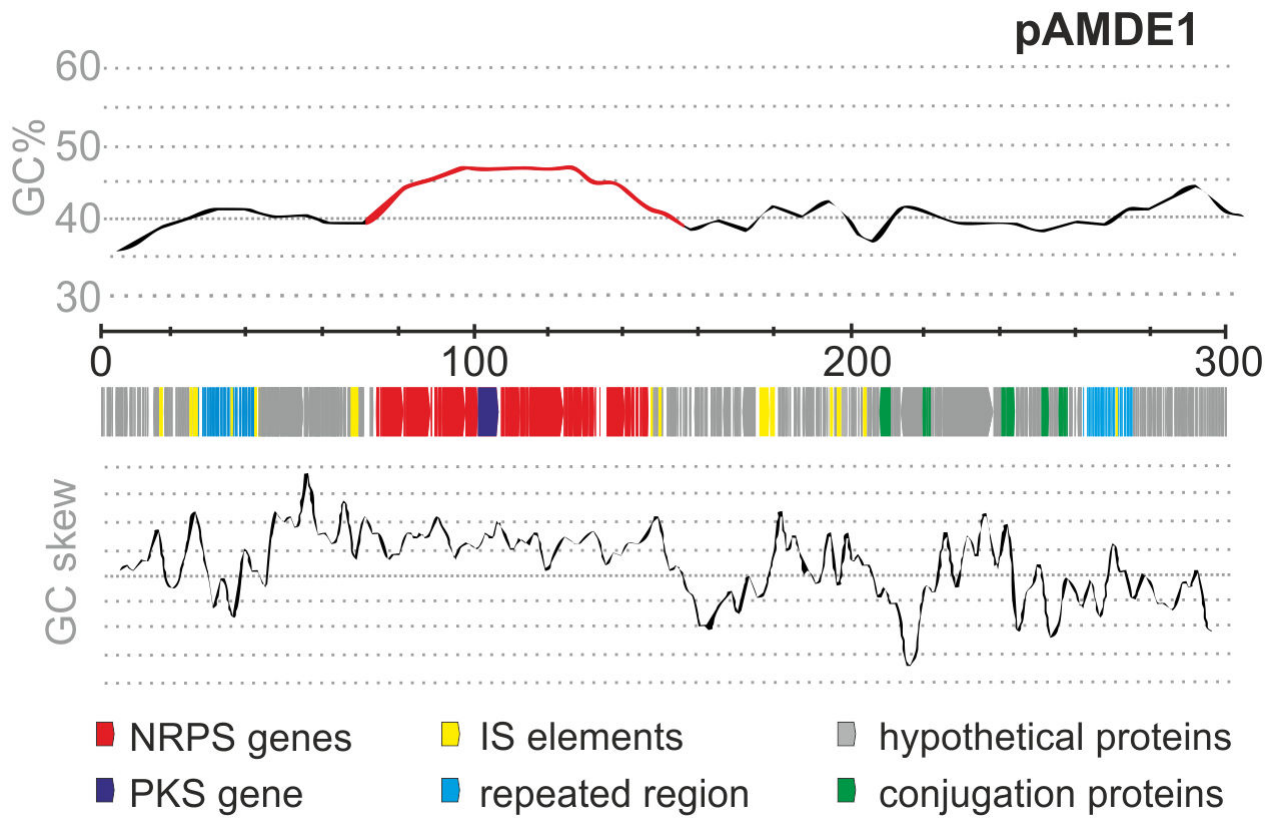
The hybrid NRPS-PKS cluster in *A* *. macleodii* . Linear representation of the plasmid pAMDE1. Scale shown in the figure is in Kilobases. GC content and GC skew are shown. The high GC region corresponding to the cluster is shown in red. Genes are color coded and a legend is provided below.

The NRPS-PKS cluster of *A* *. macleodii* was first identified in the circular, 300 kb plasmid pAMDE1 of the AltDE1 strain after genome sequencing [26]. Genome sequencing of additional *A* *. macleodii* strains isolated from the deep Ionian Sea [27] uncovered the presence of an identical plasmid in two isolates, UM7 and U4, and the insertion of the same (>99% identity) NRPS-PKS cluster within the genome of two additional strains, U7 and U8 [22]. The higher GC content of the NRPS-PKS cluster in comparison to the rest of the plasmid (Figure 1) and the presence of transposases flanking both sides of the cluster [22] are highly suggestive of a mobile element.

**Figure 2:**
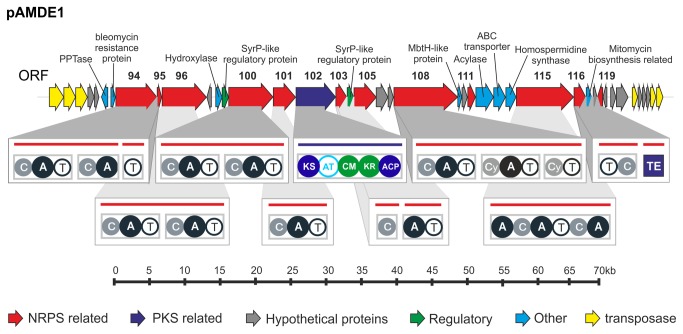
Schematic representation of the hybrid NRPS-PKS cluster of pAMDE1. Genes are colored according to the inferred function. NRPS and PKS domain architecture is shown inside boxes. NRPS and PKS gene numbers are indicated. C: Condensation domain, A: Adenylation domain, T: thiolation domain, Cy: modified condensation domain, KS: ketosynthase domain, CM: C-methyltransferase ACP: Acyl Carrier Protein domain, TE: thioesterase domain. A scale is shown below.

The hybrid NRPS-PKS cluster is 70 kb in length and encodes 28 distinct ORFS (Figure 2 and Table S1 in File S1). In the pAMDE1 cluster the genes coding for the NRPS and PKS activities are located in separate ORFs, as in the clusters of mycobactin [39] and nostopeptilide [40], rather than forming a composite gene/enzyme like in the case of nostophycin [41]. In all, one PKS and 12 NRPS genes were identified within the cluster. Other genes commonly associated with NRPS-PKS clusters, such as the phosphopantetheinyl transferase (PPTase) and the MbtH-like genes, were also identified. PPTases are responsible for the activation of the carrier proteins of fatty-acids synthases (FAS), PKSs, NRPSs and hybrids NRPS-PKS, converting them from the inactive apo-form to the active holo-form [42,43]. Since PPTases can act in different biosynthetic pathways, they are not necessarily incorporated into the cluster and may be located in other genomic regions. Another gene, MbtH, can also be found in other parts of the genome, but is not always required. Recently, however, an MbtH-like gene was shown to play a role in the activation of the adenylation domain during biosynthesis of clorobiocin [44]. A bleomycin resistance protein was also identified by homology searches. Such resistance proteins are often located within antibiotic biosynthesis clusters [45,46]. In addition, two SyrP-like regulatory proteins were also found. These are known regulators of antibiotic production. Similar proteins are found in the phytotoxin syringomycin cluster produced by *Pseudomonas syringae* [47]. Also of note is the presence of an ABC transporter protein related to a cyclic peptide transporter, indicating that the compound might be secreted.

### NRPS and PKS domains

Both NRPS and PKS biosynthesis pathways are modular with the encoding genes clustered in an assembly line fashion. The initiating module begins the synthesis of a peptidyl/acyl chain, which is subsequently elongated with other modules adding a single monomer until the final release of the entire product [48]. Within each module, for both NRPS and PKS systems, there are at least three domains: a catalytic domain for monomer selection, a carrier protein domain for holding the monomer after it is thioesterfied, and a second catalytic domain for chain elongation. In PKS biosynthesis, these domains usually take the form of an acyltransferase (AT), an acyl carrier protein (ACP), and a beta-ketoacyl synthase (KS) [49]. The pAMDE1 cluster harbors a single PKS module (Figure 2), in which a KS and ACP were readily identifiable. The AT domain did not appear to be present upon initial examination; however, subsequent analysis suggested otherwise and will be discussed below. In addition, we detected a C-methyltransferase (CM) domain and a beta-ketoacyl reductase (KR), which has been identified in Type I PKS systems previously [50]. The three core domains that constitute a functional module in NRPS biosynthesis are an adenylation (A) domain, a peptidyl carrier protein (PCP), and a condensation (C) domain [51]. The NRPS-PKS cluster described here encodes 15 NRPS modules that contain a total of 10 C domains, two modified C domains (Cy), 12 A domains, 13 PCPs containing a thiolation domain (T), and a thioesterase domain (Te).

**Figure 3:**
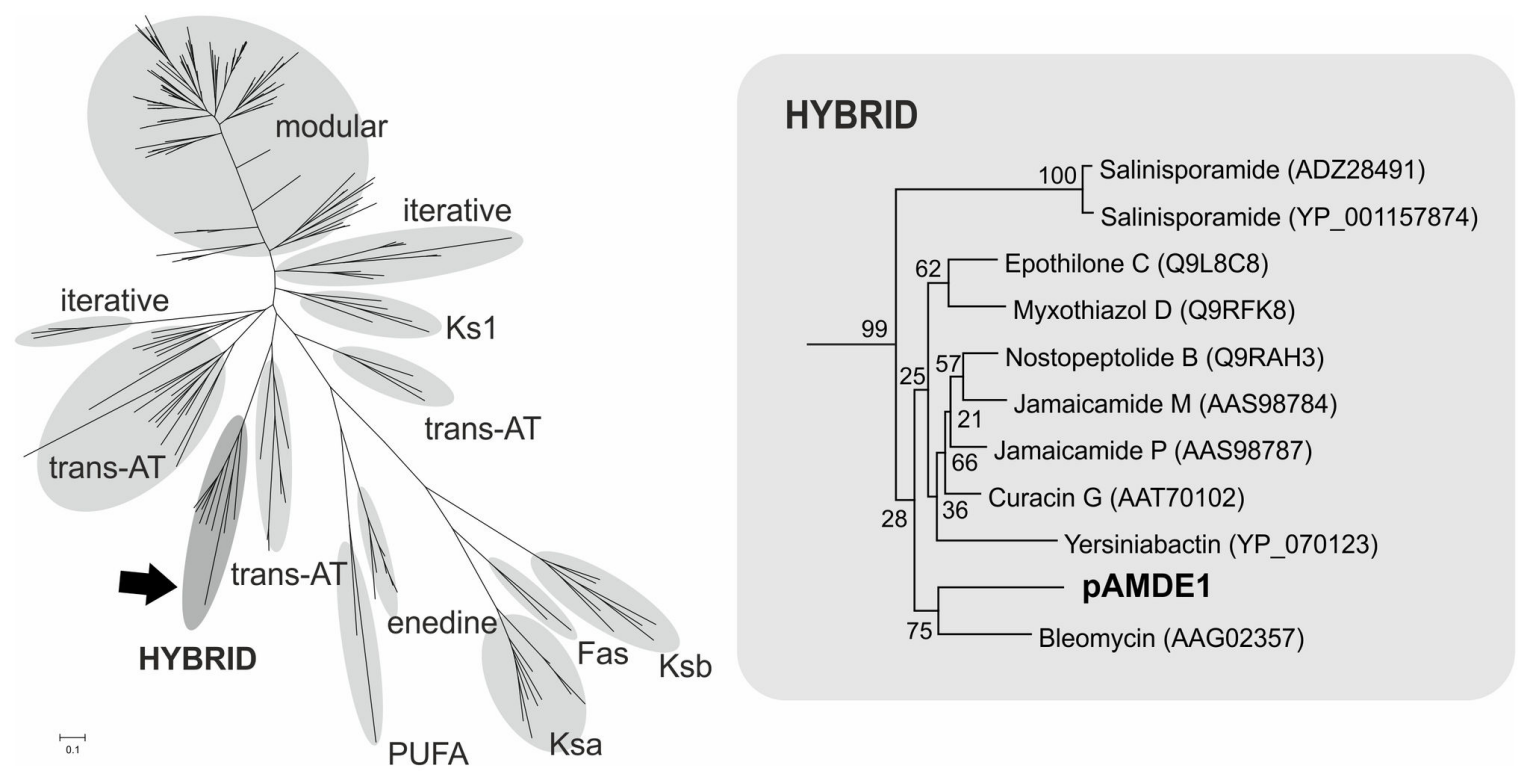
Phylogenetic tree of KS domains. All known categories of KS domains are shown in the tree. The branch containing pAMDE1 KS domain and closely related KS domains are shown in detail in the inset on the right. Bootstrap values are shown on the nodes. Ks1: KS of first module of assembly lines, Ksa: KS-alpha, Ksb: KS-beta, Fas: fatty acids, PUFA: Polyunsaturated fatty acids.

In PKS pathways, KS domains perform the essential task of catalyzing the C-C bond between each monomer. These domains present a highly conserved structure, even among KS domains from fatty acid synthases (FAS) [52]. As a result, evolutionary relationships between KS domains can be used to identify major classes of PKSs (e.g. modular, iterative, hybrid, etc.) and related compounds [53,54]. Phylogenetic analysis of the KS domain from the pAMDE1 cluster, along with KS domains of all other types of PKS genes, revealed that the KS domain described here is a hybrid domain most closely related to the KS domain of bleomycin biosynthesis modules (Figure 3).

We also analyzed the relatedness of the 14 C domains from the NRPS genes identified in the pAMDE1 cluster. Classic C domains that catalyze amino acid condensation contain an intact HHXXXDG motif [55], while deviation from this motif can indicate alternative functions [28]. Here, we detected the intact motif in seven of the 14 C domains (Table S2 in File S1). Previous studies have characterized six functional C domain groups: starter C domain (acylates the first amino acid), LCL (catalyzes peptide bond formation between 2 L-amino acids), DCL (catalyzes peptide bond formation between a D- and L- amino acid), Heterocyclization domain (CYC, catalyzes peptide bond formation and subsequent cyclization of certain amino acids), epimerization (E, flips chirality of last amino acid), dual E/C (catalyzes both epimerization and condensation) [56]. More recently, two additional groups have been proposed: modified amino acid domains (modAA, modify the incorporated amino acids) and hybrid domains (H, catalyze the condensation of an amino acid to an aminated polyketide) [33]. Using the classification described above, we were able to classify 10 of the 14 condensation domains identified in the pAMDE1 cluster (Figure S1 in File S1). The phylogenetic tree allows the identification of five LCL, two modAA, two CYC, and one H domains. In a number of branches, the pAMDE1 C domains were most closely related to bleomycin. Of the two CYC domains, one (ORF 108 C2) contained a slightly altered motif (QXXXXDX) from the conserved CYC domain motif (DXXXXDX); however, cluster analysis still clustered this domain with the CYC domains. One C-domain also clustered with DCL domains (as predicted by the NAPDOS server [33]), suggesting the incorporation of a D-amino acid into the final product. However, the pAMDE1 cluster did not present any evidence of any epimerization domains that are essential for this incorporation [56,57,58]. Classification of C-domains as DCL may be misleading as some C-domains, especially those related to glycopeptide biosynthesis are actually LCL domains hypothesized to be derived from DCL domains [56]. This appears to be the case in the prediction of a DCL domain in bleomycin by the NAPDOS server, when the gene cluster coding for bleomycin does not display any evidence for an epimerization domain. Moreover, examination of the phylogenetic tree of the C-domains reveals that the domains classified as DCL by the NAPDOS server, also do not present high bootstrap values. For example, both the bleomycin and the pAMDE1 C-domain are part of a branch that has weak bootstrap support (only 20%). In comparison, several other predictions have much better support e.g CYC domain (100%), the modAA (100%), LCL (81%). Taken together, this suggests that D-amino acids are not incorporated into the final product of the pAMDE1 cluster.

The A domains in the NRPS proteins are responsible for recruiting amino-acid monomers to be incorporated in to the final product produced by the cluster. In many cases, the sequential order of the A domains determines the sequence of the final peptide. As biochemical specificities of several A domains have been characterized [59], it has become possible to use sequence dependent searches and defined sequence motifs to predict the specificity of novel A domains [60]. The predicted specificities of the pAMDE1 eleven A domains were predicted to be Ser, Lys, Leu, L-Asn, L-His, Phe, Gln, β-Ala, L-Cys(2), L-Ser and L-Asn, while two could not be predicted (Table 1). We also identified the domain from which the specificity of our A domain was predicted. From the 13 A domains, six could be identified by their similarity to the adenylation domains from bleomycin, one was more similar to that of bacitracin, and one was more similar to the A domain of the NRP calcium-dependent antibiotic from *Streptomyces coelicolor*. The others were not identified. This additionally suggests that this cluster is closely related to the one of bleomycin.

**Table 1:** Amino acid specificity of A domain of pAMDE1 based on their motifs.

**A DOMAIN**	**235**	**236**	**239**	**278**	**299**	**301**	**322**	**330**	**331**	**517**	**SUBSTRATE**
pAMDE1-94-A1	D	V	W	H	F	S	L	V	D	-	Ser
pAMDE1-94-A2	D	I	E	S	V	G	T	C	Y	-	Lys
pAMDE1-96-A1	D	A	H	F	F	S	Y	V	V	K	Leu
pAMDE1-96-A2	I	R	W	V	F	S	L	S	D	K	unkn
pAMDE1-100-A1	D	L	T	K	V	G	E	V	G	K	L-Asn
pAMDE1-100-A2	D	S	A	L	I	A	E	V	W	K	L-His
pAMDE1-101-A1	D	V	F	T	Y	A	L	V	Y	K	Phe
pAMDE1-105-A1	D	A	W	Q	V	G	V	I	H	K	Gln
pAMDE1-108-A1	V	D	A	T	V	S	I	A	D	K	β-Ala
pAMDE1-108-A2	D	L	Y	N	L	S	L	I	W	-	L-Cys(2)
pAMDE1-115-A1	D	Q	V	G	F	G	A	L	V	K	unkn
pAMDE1-115-A2	D	V	W	H	I	S	L	I	D	K	L-Ser
pAMDE1-115-A3	D	M	T	K	L	G	E	V	G	K	L-Asn

The numbers at the top are residue identifiers as described in NRPSpredictor2 and SBSPKS servers. L-Cys(2): Two motifs code for Cysteine, this is the second one. Unkn: unknown

### Comparative analysis of the biosynthetic cluster of pAMDE1 and bleomycin clusters

The close phylogenetic relationship of the PKS (i.e., KS) and NRPS (i.e., C and A) domains described here with bleomycin domains prompted us to perform a comparison of the AltDE1 cluster with the hybrid NRPS-PKS cluster of bleomycin (BLM) [46] and two other members of the bleomycin family: tallysomycin (TLM) [61] and zorbamycin (ZBM) [62] (Figure S2 in File S1). Although alignment of the four clusters based on sequence similarity revealed large differences (Figure S3 in File S1), a more detailed comparison of the sequences showed that the bleomycin family sequences were consistently the best matches for homologous genes (Table S3 in File S1). For example, ORF 108 of pAMDE1 was closely related to the genes *blmIV*, *tlmIV* and *zbmIV* from the bleomycin, tallysomycin and zorbamycin clusters, respectively. Using this information, we were able to identify 20 genes out of 27 in the pAMDE1 cluster that have homologous genes in the bleomycin related clusters. The comparisons suggest that the pAMDE1 NRPS-PKS cluster most closely resembles that of ZBM, which contained all 20 genes, while BLM and TLM contained 16 and 17, respectively. One AltDE1 gene (ORF 112) was unique to ZBM and annotated as an acylase, which hydrolyzes acylated amino acids. Three additional pAMDE1 genes were identified in both ZBM and TLM, but not BLM. These include ORF 93, a binding protein, implicated in resistance to the bleomycin family antibiotics; ORF 98, a hydroxylase; and ORF 113, a multidrug transporter. The SyrP-like regulators associated with pAMDE1 (ORFs 99 and 104) are similar to that of all three bleomycin family compounds. However, despite their similarities at the structural level, the complete lack of genes involved in sugar biosynthesis in the pAMDE1 cluster clearly indicates that the final compound produced by AltDE1, unlike the bleomycin family compounds, is not a glycopeptide (Figure S3 in File S1).

**Figure 4:**
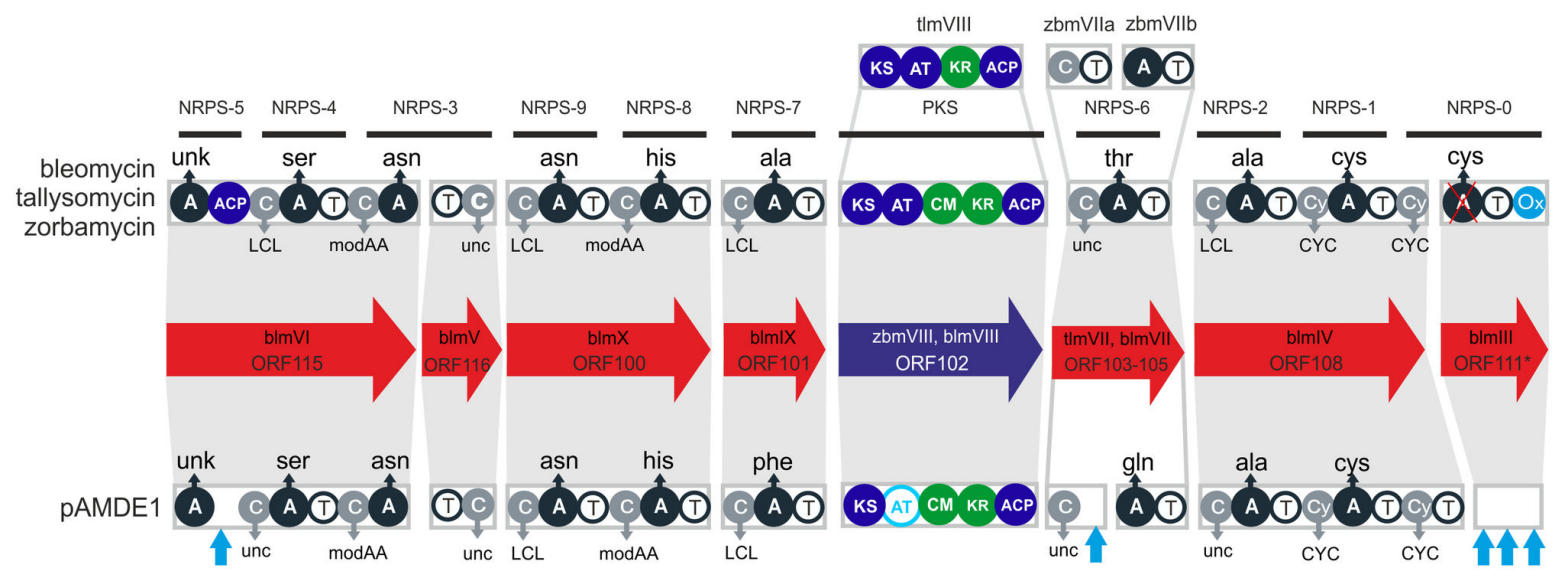
Comparison of the protein domains of pAMDE1 to bleomycin related clusters. NRPS genes are represented in red and the PKS gene in blue. Within the arrows the names of the ORF in pAMDE1 and the homologs in the bleomycin or the other clusters are indicated. The protein domains are shown inside the boxes. In addition, functional classification is shown for condensation domains (C) and the substrate amino acid for each adenylation domain (A). The modules (NRPS-0 to NRPS-9) described for the bleomycin compounds are represented by the black lines above the domains. Light blue arrows indicate putative missing domains in pAMDE1 in comparison to bleomycin. AT and KR domains are also shown in light blue. Blm genes: bleomycin, zbm genes: zorbamycin, tlm genes: tallyzomycin. LCL: catalyzes peptide bond formation between 2 L-amino acids, CYC: heterocyclization domain, modAA: modify the incorporated amino acids, unc: unclassified. A non-functional A-domain is marked with a red-cross.

A comparison of the protein domain architectures showed that the overall arrangement of the pAMDE1 NRPS-PKS and bleomycin family megasynthases is well-conserved (Figure 4), providing further support for a relationship between this cluster and that of BLM, TLM and ZBM. For example, ORF 100 of pAMDE1 is homologous to the bleomycin family genes *blmX*, *tlmX* and *zbmX*, and it encodes the same modules containing identical domains (i.e., CATCAT) with the same functional classification (e.g., the first C domain is an LCL, while the second C domain is a modAA). The two CYC domains located in ORF 108, as well as *blmIV*, *tlmIV*, and *zbmIV*, also highlight this conserved arrangement.

Comparative analysis of the bleomycin related compounds has highlighted the complexity of their biosynthesis [28]. The analysis of pAMDE1 C domains points towards a very similar functionality to its counterparts in bleomycin biosynthesis (Figure 4). A more detailed analysis of the eight C domain motifs revealed two (ORF 100-C2 and 116-C2) identical C domains (Table S4 in File S1). Four domains (ORF 101-C1, 103-C1, and 108-C1, 115-C1) contained slight alterations, while the final two (ORF 100-C1 and 115-C2) exhibited significant differences. A domains, which exhibit specificity for a single amino acid also showed similarities between pAMDE1 and the bleomycin domains (Figure 4). The A domains of bleomycin related compounds load the following amino acids Ser-Asn-Asn-His-Ala-Thr-Ala-Cys to form the backbone structure, which differs from that of pAMDE1 by two amino acids (Ser-Asn-Asn-His-Phe-Gln-Ala-Cys). Adenylation domain motif comparisons also reflect a high degree of similarity to bleomycin (Table S5 in File S1). For the six A domains that confer the same specificity between pAMDE1 and the bleomycin family cluster, the motifs are either identical or differ by a single mismatch. These NRPS domain similarities provide strong support for a relationship with the bleomycin family.

In the case of the PKS gene, the KS, CM, KR and ACP domains were readily identified, while the AT domain was not detected initially. However, comparison of the pAMDE1 cluster with that of the bleomycin family clusters suggested that the AT domain might also be present (Figure 4). The region of the protein where the AT domain was present (suggested by multiple alignments) showed very weak similarity with known AT domains. However, a 3D-structure prediction [31] confidently identified the AT domains of several fatty acid synthases as the best templates for this region of the protein. A structural alignment of known acyltransferase domains with the AT domain sequences of the bleomycin family indicated the presence of several conserved residues indicating close structural similarity (Figure S4 in File S1). Even so, examination of amino acids involved in the catalysis as described before [63] showed that only one of them was fully conserved in this alignment. The catalytic serine residue was also not conserved. However, this particular residue is missing in the zorbamycin AT domain as well. This suggests that this particular AT domain in the pAMDE1 PKS gene is divergent, although still structurally recognizable, has an as yet unknown catalytic site (Figure S4 in File S1).

### Phleomycin resistance

**Figure 5:**
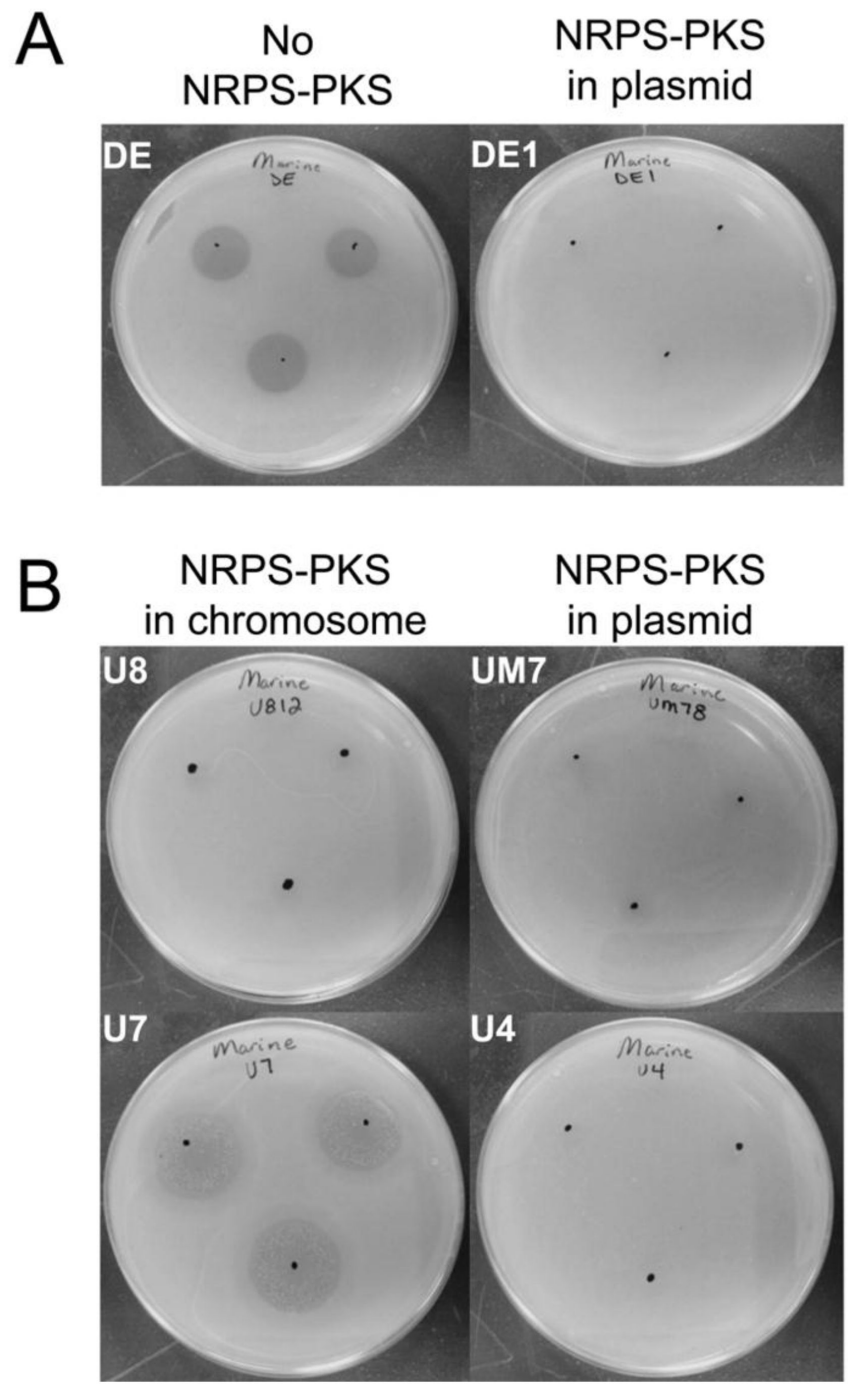
Phleomycin resistance in *Alteromonas* *macleodii* strains. A) Phleomycin assay of AltDE (DE, lacking the NRPS-PKS cluster) and AltDE1 (DE1, harboring the NRPS-PKS cluster in the plasmid pAMDE1) on marine agar plates. B) Phleomycin assay of strains U7 and U8 (both harbor the NRPS-PKS cluster in their chromosome) and UM7 and U4 (both harbor the NRPS-PKS cluster in their plasmids).

The similarity between the NRPS-PKS cluster described here to the bleomycin biosynthesis cluster, in addition to the presence of a gene with some similarity (~53% similarity) to known bleomycin resistance genes (i.e., *tlmA* and *zbmA*), led us to investigate the susceptibility of seven *A* *. macleodii* strains to the bleomycin family. It is common for microorganisms that produce bleomycin related antibiotics to carry resistance genes that confer self-resistance [45,61,62], and we wondered if the bleomycin resistance protein of the NRPS-PKS cluster, in spite of its divergence, would still confer resistance to phleomycin (a bleomycin related compound) [64]. The two strains from the Adriatic Sea (AltDE and AltDE1) differed, with AltDE exhibiting susceptibility, while AltDE1 was clearly resistant (Figure 5A). The strains from the Ionian Sea (Figure 5B) revealed a similar pattern with UM7 and U4 being resistant. Interestingly, of the two strains with the NRPS-PKS cluster inserted into the chromosome, one (U8) was resistant and one (U7) was partially susceptible (Figure 5B). The susceptibility phenotype of U7 differed from AltDE, which does not contain an NRPS-PKS cluster, in that U7 exhibited some growth (i.e., individual colonies) within the clearing zone (Figure 5B), while AltDE exhibited no growth within the clearing zone (Figure 5A). We obtained consistent results at two temperatures (13 and 25°C) and two dosages (50 and 100 µg) of antibiotic. These results show that the bleomycin resistance protein confers complete or at least partial resistance to phleomycin and argue that the final compound of the NRPS-PKS cluster described here is structurally similar to the bleomycin family of antibiotics.

### NRPS-PKS cluster expression

**Figure 6:**
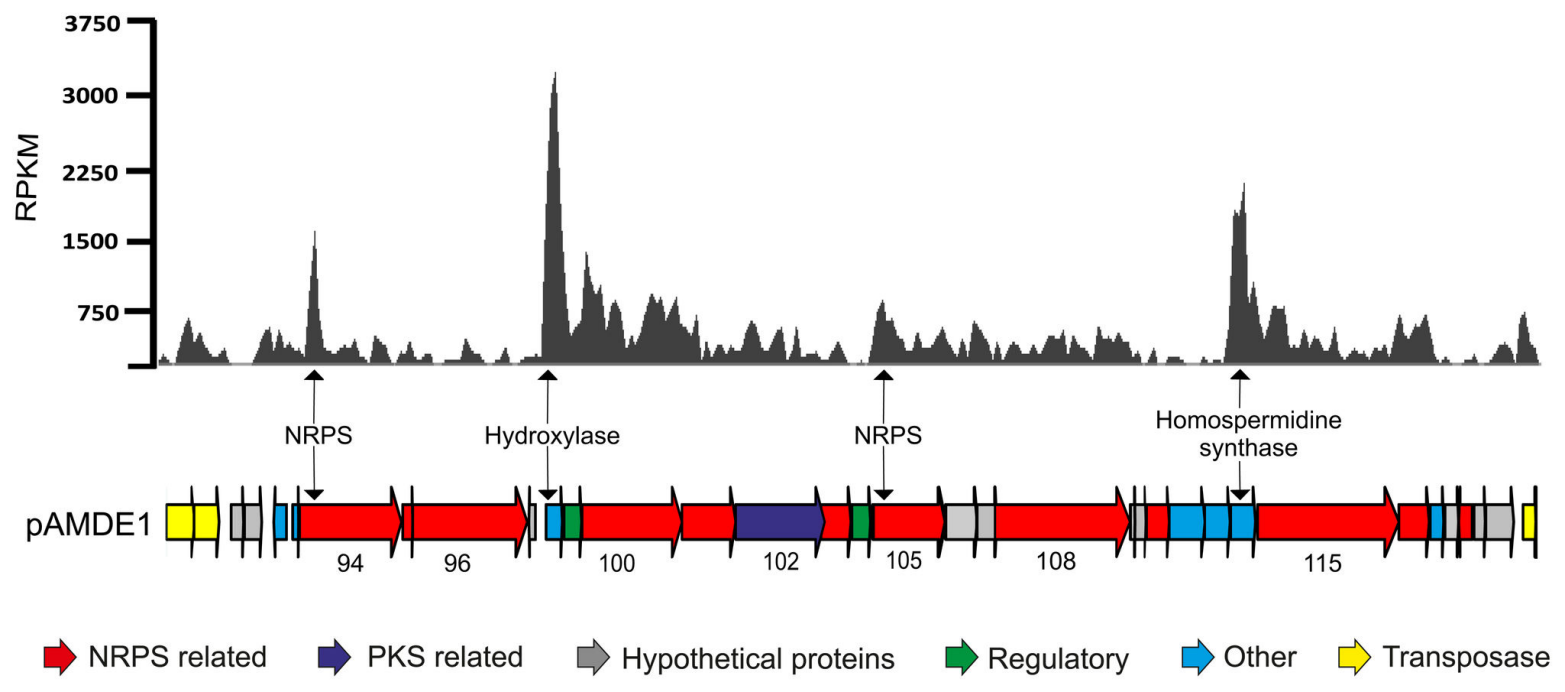
Gene expression of the NRPS-PKS cluster. The graph shows the estimated gene expression using RPKM values associated with the NRPS-PKS cluster of pAMDE1. Genes are colored according to the inferred function. The numbers below some of the box arrows correspond to ORF numbers for that particular gene.

Although the presence of a NRPS-PKS cluster is indicative of secondary metabolite production, gene expression is a prerequisite. There are cases in which cryptic PKS genes have been identified by genomic methods, yet expression is not detected [22]. This is not unusual, as laboratory culture methods likely are poor approximations of natural conditions in which such products are expressed. To determine if the cluster observed in AltDE1 is expressed, we performed RNA sequencing on mid-exponential phase AltDE1 grown in MB. The transcriptional analysis revealed that almost the entire NRPS-PKS cluster is expressed (Figure 6). In addition, we validated the expression of three genes (two NRPS and one PKS) that span the entire cluster. One NRPS gene (ORF 94) is located at the beginning of the cluster, the second (ORF 108) at the end, and the PKS gene (ORF 102) resides in the middle (Figure 2). PCR amplification of cDNA revealed expression of all three genes (data not shown). These data provide strong support that the complete NRPS-PKS cluster is not cryptic.

Although we provide evidence that several genes of this cluster are expressed during standard culturing conditions, there are many factors that likely affect the subsequent activity of this compound. For example, nutrient availability (both type and concentration) is known to affect the production of antibiotics, including specific carbon sources that can affect either the transcription or translation of specific genes [65,66]. Environmental conditions, such as temperature [67] and pH [68], can also affect antibiotic activity, as can the presence of an antagonist [69]. We performed preliminary bioassays using the supernatant of AltDE1 and did not observe any obvious activity against closely related strains of *A* *. macleodii* , *Bacillus subtilis*, or the fungus *Saccharomyces cerevisiae* (data not shown). Future studies will need to focus on a variety of culturing conditions in order to identify the compound produced via this newly described hybrid NRPS-PKS cluster and its activity.

## Conclusions

In this study, we describe in detail a novel, mobile, hybrid NRPS-PKS cluster first identified in the plasmid of the *Alteromonas* *macleodii* strain AltDE1 and provide evidence that this cluster is related to that of the bleomycin glycopeptide antibiotic family. Transcriptomic data for the pAMDE1 cluster provide strong support that the NRPS-PKS cluster is being expressed. The genes in the cluster coding for the NRPS and PKS activities are located in separate ORFs, and further analysis of the PKS protein indicates that this particular PKS gene carries a novel acyltransferase domain. Despite the similarities of the protein domain architectures of pAMDE1 to those from the bleomycin family, the lack of genes involved in sugar biosynthesis in the pAMDE1 cluster suggests that the final compound produced by AltDE1 is not a glycopeptide. The similarities in the predicted specificities of the adenylation domains of pAMDE1and known specificities of bleomycin cluster adenylation domains suggested that the final compound of pAMDE1 has a bleomycin-like backbone. This deduced similarity was also manifest in the phleomycin resistance conferred by the presence of the cluster in the genome of *A* *. macleodii* strains, while strains naturally deficient in this cluster were clearly susceptible. The differential presence of this cluster in different *Alteromonas* strains isolated from the same geographical location provides clues to possible competitive behavior even within these different strains and with other marine microbes in a particle associated and r-strategist-like lifestyle. It is likely that the presence of such a cluster and production of this secondary metabolite provides an advantage to *A* *. macleodii* assisting it in becoming one of the most abundant microbes in oceanic blooms.

## Supporting Information

File S1 **Supporting Files**.
Table S1, List of all genes and function for predicted genes of the pAMDE1 cluster. Table S2, C domain motifs identified in the pAMDE1 gene cluster. Amino acids that differ from the classical C domain motif are shown in red. The numbers at the top are residue identifiers as described in SBSPKS server. Table S3, Gene comparison of pAMDE1 to BLM, TLM and ZBM clusters. Table S4, C domain motifs of pAMDE1 in comparison to the BLM, TLM and ZBM motifs. Amino acids that differ from the classical C domain motif are shown in red. The numbers at the top are residue identifiers as described in SBSPKS server. Table S5, A domain motifs and amino acid specificity of pAMDE1 in comparison to that of BLM, TLM and ZBM. Amino acids that differ from the BLM, TLM and ZBM domain motifs are shown in red. The numbers at the top are residue identifiers as described in NRPSpredictor2 and SBSPKS server. Figure S1, phylogenetic tree of C domains. All known categories of C domains are shown. C domains of pAMDE1 are highlighted in bold. LCL: catalyzes peptide bond formation between 2 L-amino acids, DCL: catalyzes peptide bond formation between a D- and L- amino acid, CYC: heterocyclization domain, epimerization: flips chirality of last amino acid, dual E/C: catalyze both epimerization and condensation, modAA: modify the incorporated amino acids, H: hybrid, UNC: unclassified. Bootstrap values are shown on the branches. Figure S2, bleomycin family of antitumor antibiotics. A) Structure of BLM, TLM and ZBM. Structural differences are highlighted with red arrows. B) Gene cluster representation and protein domains of bleomycin (blm), tallysomycin (tlm) and zorbamycin (zbm). The protein domains are shown inside the boxes. The modules (NRPS-0 to NRPS-9) described for the bleomycin compounds are represented by the black lines above the domains. The NRPS genes are represented by red arrows, and the PKS gene is represented by a blue arrow. Figure S3, Cluster comparison of pAMDE1 to BLM, TLM and ZBM. All genes are colored according to the inferred function. Although all-versus-all comparisons (using BLASTn) were made, only selected pairwise comparisons are shown for clarity. The level of similarity between different contigs is indicated in the legend on the left. The name of the genes of pAMDE1 (represented by ORFs) and zorbamycin are shown. Figure S4, Structural alignment. Acyltransferase domains from four sequences of the bleomycin family and three known protein structures (PDB codes: 1MLA, 3QAT, 2JFD) are shown in the alignment. The alignment was created using the PROMALS3D web server. The first line in each block shows conservation indices for positions with a conservation index above 2. The last two lines show consensus amino acid sequence (Consensus_aa) and consensus predicted secondary structures (Consensus_ss). Consensus amino acid symbols are: conserved amino acids are in bold and uppercase letters; aliphatic: l; aromatic: @; hydrophobic: h; alcohol: o; polar residues: p; tiny: t; small: s; bulky residues: b; positively charged: +; negatively charged: -; charged: c. Known active site residues in the protein structure of 1MLA are indicated by a orange star symbol on top.
(PDF)
