# Supplementary material for: A Hybrid NRPS-PKS Gene Cluster Related to the Bleomycin Family of Antitumor Antibiotics in Alteromonas macleodii Strains
Source: PLoS One. 2013 Sep 19;8(9):e76021. doi: 10.1371/journal.pone.0076021 (PMC3777966; doi:10.1371/journal.pone.0076021)
Supplement: File S1 — Supporting Files. Table S1, List of all genes and function for predicted genes of the pAMDE1 cluster. Table S2, C domain motifs identified in the pAMDE1 gene cluster. Amino acids that differ from the classical C domain motif are shown in red. The numbers at the top are residue identifiers as described in SBSPKS server. Table S3, Gene comparison of pAMDE1 to BLM, TLM and ZBM clusters. Table S4, C domain motifs of pAMDE1 in comparison to the BLM, TLM and ZBM motifs. Amino acids that differ from the classical C domain motif are shown in red. The numbers at the top are residue identifiers as described in SBSPKS server. Table S5, A domain motifs and amino acid specificity of pAMDE1 in comparison to that of BLM, TLM and ZBM. Amino acids that differ from the BLM, TLM and ZBM domain motifs are shown in red. The numbers at the top are residue identifiers as described in NRPSpredictor2 and SBSPKS server. Figure S1, phylogenetic tree of C domains. All known categories of C domains are shown. C domains of pAMDE1 are highlighted in bold. LCL: catalyzes peptide bond formation between 2 L-amino acids, DCL: catalyzes peptide bond formation between a D- and L- amino acid, CYC: heterocyclization domain, epimerization: flips chirality of last amino acid, dual E/C: catalyze both epimerization and condensation, modAA: modify the incorporated amino acids, H: hybrid, UNC: unclassified. Bootstrap values are shown on the branches. Figure S2, bleomycin family of antitumor antibiotics. A) Structure of BLM, TLM and ZBM. Structural differences are highlighted with red arrows. B) Gene cluster representation and protein domains of bleomycin (blm), tallysomycin (tlm) and zorbamycin (zbm). The protein domains are shown inside the boxes. The modules (NRPS-0 to NRPS-9) described for the bleomycin compounds are represented by the black lines above the domains. The NRPS genes are represented by red arrows, and the PKS gene is represented by a blue arrow. Figure S3, Cluster comparison of pAMDE1 to BLM, [file pone.0076021.s001.pdf]

File S1

A hybrid NRPS-PKS gene cluster related to the bleomycin family of antitumor antibiotics in *Alteromonas macleodii* strains

Carolina Megumi Mizuno[1], Nikole E. Kimes[1], Mario López-Pérez[1], Eva Ausó[1], Francisco Rodriguez-Valera[1*] and Rohit Ghai[1]

[1]Evolutionary Genomics Group, Departamento de Producción Vegetal y Microbiologia, Universidad Miguel Hernández, San Juan de Alicante, Alicante, Spain

*****frvalera@umh.es

Table S1. List of all genes and function for predicted genes of the pAMDE1 cluster.

ORF identifier	length (aa)	proposed function (domain)	description of blast first hit [taxonomy]	similarity / identity
ORF86	*274*	*TnsA endonuclease*	*TnsA endonuclease [Escherichia coli]*	*73.16 / 58.82*
ORF87	*716*	*TnsB transposase*	*TnsB transposase [Escherichia coli]*	*78.15 / 65.69*
ORF88	*481*	*TnsC ATPase*	*TnsC ATPase [Escherichia coli]*	*77.45 / 62.84*
ORF89	*470*	*Hypothetical protein*	*conserved hypothetical protein [Vibrio cholerae RC385]*	*67.66 / 47.87*
ORF90	*327*	*Unknown protein*	-	-
ORF91	*40*	*Hypothetical protein*	*hypothetical protein MED222_16571 [Vibrio sp. MED222]*	*88.57 / 88.57*
ORF92	244	Phosphopantetheinyl transferase	Phosphopantetheinyl transferase component of siderophore synthetase [Pseudoalteromonas tunicata D2]	62.18 / 45.38
ORF93	114	Sh Ble binding protein	Glyoxalase/bleomycin resistance protein/dioxygenase [Ralstonia pickettii 12J]	65.09 / 46.23
ORF94	1960	NRPS (CATCA)	amino acid adenylation domain-containing protein [Nostoc punctiforme PCC 73102]	55.87 / 36.02
ORF95	196	NRPS (T)	non-ribosomal peptide synthetase, terminal component [Pseudoalteromonas tunicata D2]	56.45 / 33.33
ORF96	2200	NRPS (CATCAT)	non-ribosomal peptide synthase [Nostoc sp. 'Peltigera membranacea cyanobiont']	52.06 / 33.83
ORF97	147	Hypothetical protein	hypothetical protein VeAt4_02692, partial [Verminephrobacter sp. At4]	56.84 / 35.79
ORF98	309	beta-hydroxylase	beta-hydroxylase [Streptomyces verticillus]	56.8 / 39.8
ORF99	329	SyrP-like protein, oxigenase	Taurine catabolism dioxygenase TauD/TfdA [Burkholderia thailandensis MSMB43]	54.1 / 37.7
ORF100	2096	NRPS (CATCAT)	TlmX [Streptoalloteichus hindustanus]	53.74 / 35.61
ORF101	1126	NRPS (CAT)	amino acid adenylation domain-containing protein [Nostoc punctiforme PCC 73102]	47.48 / 30.19
ORF102	1861	PKS (KS-CM-ACP)	beta-ketoacyl synthase [Streptococcus macacae NCTC 11558]	59.44 / 43.54
ORF103	537	NRPS (C)	amino acid adenylation protein [Chitinophaga pinensis DSM 2588]	44.26 / 26.84
ORF104	354	SyrP-like protein, oxigenase	oxygenase [Streptomyces flavoviridis]	53.24 / 38.13
ORF105	1026	NRPS (AT)	peptide synthetase [Nostoc sp. PCC 7120]	48.28 / 28.75
ORF106	511	Hypothetical protein	hypothetical protein RICGR_0543 [Rickettsiella grylli]	40.52 / 23.42
ORF107	300	Hypothetical protein	hypothetical protein HCH_02806 [Hahella chejuensis KCTC 2396]	53.06 / 37.07
ORF108	3066	NRPS (CATCyATCyT)	nonribosomal peptide synthase family [Microcoleus chthonoplastes PCC 7420]	48.51 / 30.73
ORF109	79	MbtH-like protein	hypothetical protein sce3255 [Sorangium cellulosum 'So ce 56']	74.63 / 55.22
ORF110	280	Hypothetical protein	hypothetical protein Mevan_0493 [Methanococcus vannielii SB]	47.16 / 29.55

ORF111	507	NRPS	amino acid adenylation domain-containing protein [Verminephrobacter sp. At4]	43.29 / 21.65
ORF112	793	Acylase	penicillin amidase [Teredinibacter turnerae T7901]	53.9 / 37.4
ORF113	562	ABC multi-drug transport	cyclic peptide transporter [Teredinibacter turnerae T7901]	62.61 / 41.56
ORF114	474	Homospermidine synthase	homospermidine synthase [Shewanella amazonensis SB2B]	59.11 / 39.41
ORF115	2721	NRPS (ACATCA)	TlmVI [Streptoalloteichus hindustanus]	47.33 / 29.24
ORF116	580	NRPS (TC)	putative multi-domain non-ribosomal peptide synthetase [Candidatus Nitrospira defluvii]	45.16 / 28.52
ORF117	260	related to mitomycin antibiotics	protein involved in biosynthesis of mitomycin antibiotics/polyketide fumonisin [Rheinheimera sp. A13L]	73.12 / 53.36
ORF119	253	Thioesterase	predicted thioesterase involved in non-ribosomal peptide biosynthesis [Pseudoalteromonas tunicata D2]	77.58 / 57.85
ORF120	*32*	*Unknown protein*	-	-
ORF121	*213*	*Hypothetical protein*	*hypothetical protein Rhein_3302 [Rheinheimera sp. A13L]*	*85.8/72.78*
ORF122	*534*	*Hypothetical protein*	*hypothetical protein Rhein_3302 [Rheinheimera sp. A13L]*	*83.52/67.98*
ORF123	*239*	*Integrase*	*integrase [Burkholderia glumae BGR1]*	*51.85/31.48*
ORF124	*95*	*Hypothetical protein*	*hypothetical protein PFLU5696 [Pseudomonas fluorescens SBW25]*	*73.68/58.95*
ORF125	*68*	*Unknown protein*	-	-
ORF126	*98*	*Unknown protein*	-	-
ORF127	*159*	*Unknown protein*	-	*68.46/53.05*
ORF128	*309*	*Transposase*	*transposase [Vibrio anguillarum 775]*	*404 / 68.46*

*Genes flanking the NRPS-PKS hybrid cluster are also shown and are italicized.

Table S2. C domain motifs identified in the pAMDE1 gene cluster. Amino acids that differ from the classical C domain motif are shown in red. The numbers at the top are residue identifiers as described in SBSPKS server.

	conserved motif						
**DOMAIN**	**H**	**H**	**Xa**	**X**	**X**	**D**	**G**
pAMDE1-94-C1	**H**	**H**	I	I	A	**D**	**G**
pAMDE1-94-C2	**H**	**H**	I	G	C	**D**	**G**
pAMDE1-96-C1	**H**	**H**	I	A	V	**D**	**G**
pAMDE1-96-C2	**H**	**H**	I	A	V	**D**	**G**
pAMDE1-100-C1	**P**	**A**	M	F	G	**D**	**K**
pAMDE1-100-C2	**H**	**H**	E	I	I	**D**	**G**
pAMDE1-101-C1	**H**	**H**	L	I	I	**D**	**E**
pAMDE1-103-C1	**H**	**H**	I	C	S	**D**	**G**
pAMDE1-108-C1	**H**	**H**	L	I	S	**D**	**A**
pAMDE1-108-C2	**Q**	**M**	I	M	I	**D**	**L**
pAMDE1-108-C3	**D**	**L**	I	V	A	**D**	**T**
pAMDE1-115-C1	**H**	**H**	A	I	C	**D**	**F**
pAMDE1-115-C2	**-**	**E**	V	S	M	**D**	**G**
pAMDE1-116-C1	**H**	**H**	L	I	L	**D**	**G**

Table S3. Gene comparison of pAMDE1 to BLM, TLM and ZBM clusters.

pAMDE1	length (aa)	Streptomyces verticillus ATCC15003	similarity / identity	Streptoalloteichus hindustanus E465-4 ATCC 31158	similarity / identity	Streptomyces flavoridis ATCC 21892	similarity / identity
ORF93	114	-	-	**TlmA Sh Ble binding protein (ABL74956.1)**	**53.1 / 33.63**	**zbmA zbm binding protein (ACG60763.1)**	**53.04 / 34.78**
ORF94	1960	NRPS11-10 (AAG02349.1)	48.29 / 30.14	TlmVI NRPS5-4 (ABL74940.1)	46.75 / 28.91	zbmVI NRPS5-4 (ACG60776.1)	43.39 / 26.55
ORF95	196	blmX NRPS9-8 (AAG02355.1)	52.94 / 30	TlmX NRPS9-8 (ABL74936.1)	51.76 / 31.76	zbmX NRPS9-8 (ACG60782.1)	51.2 / 31.93
ORF96	2200	NRPS11-10 (AAG02349.1)	46.38 / 31.41	TlmIV NRPS2-1 (ABL74945.1)	44.62 / 27.55	ZbmIV NRPS2-1 (ACG60772.1)	42.58 / 26.47
ORF98	309	-	-	**Tlm Orf10 hydroxylase (ABL74955.1)**	**57.48 / 37.41**	**Zbm-Orf30 hydroxylase (ACG60751.1)**	**59.03 / 40.28**
ORF99	329	SyrP-like protein (AAG02342.1)	52.1 / 36.01	TlmR3 SyrP-like regulatory protein (ABL74947.1)	53.23 / 34.84	Zbm-Orf19 oxygenase (ACG60747.1)	53.26 / 37.68
ORF100	2096	blmX NRPS9-8 (AAG02355.1)	52.43 / 35.21	TlmX NRPS9-8 (ABL74936.1)	53.74 / 35.61	zbmX NRPS9-8 (ACG60782.1)	53.04 / 35.78
ORF101	1126	blm IX NRPS7 (AAG02356.1)	41.96 / 25.78	TlmIX NRPS7 (ABL74937.1)	44.41 / 26.35	zbmIX NRPS7 (ACG60773.1)	42.75 / 25.02
ORF102	1861	blm VIII PKS (AAG02357.1)	52.62 / 38.12	TlmVIII PKS (ABL74938.1)	53.21 / 37.87	zbmVIII PKS (ACG60781.1)	42.85 / 27.97
ORF103	537	blmVII NRPS6 (AAG02358.1)	38.36 / 22.64	TlmVII NRPS6 (ABL74939.1)	38.82 / 23.73	ZbmIV NRPS2-1 (ACG60772.1)	42.52 / 25.7
ORF104	354	SyrP-like protein (AAG02342.1)	51.19 / 34.81	TlmR3 SyrP-like regulatory protein (ABL74947.1)	52.96 / 34.84	Zbm-Orf19 oxygenase (ACG60747.1)	53.24 / 38.13
ORF105	1026	blmVII NRPS6 (AAG02358.1)	43.31 / 26.87	TlmVI NRPS5-4 (ABL74940.1)	44.73 / 26.81	ZbmIV NRPS2-1 (ACG60772.1)	46.9 / 30.09
ORF108	3066	blmIV NRPS2-1 (AAG02364.1)	46.84 / 30.67	TlmIV NRPS2-1 (ABL74945.1)	48.6 / 31.02	ZbmIV NRPS2-1 (ACG60772.1)	46.29 / 30.51
ORF109	79	MbtH-like protein (AAG02368.1)	76.79 / 57.14	Tlm Orf16 MbtH-like protein (ABL74949.1)	77.42 / 54.84	Zbm-Orf21 unknown (ACG60748.1)	77.78 / 59.26
ORF111	507	NRPS11-10 (AAG02349.1)	37.28 / 22.78	-	-	ZbmIV NRPS2-1 (ACG60772.1)	45.34 / 28.57

ORF112	793	-	-	-	-	**Zbm-Orf4 acylase (ACG60745.1)**	**39.1 / 23.08**
ORF113	562	-	-	**TlmT ABC multi-drug transport (ABL74935.1)**	**43.27 / 20.18**	**Zbm-Orf38 ABC transporter (ACG60758.1)**	**45.65 / 26.09**
ORF115	2721	blmVI NRPS5-4-3 (AAG02359.1)	47.12 / 31.66	TlmVI NRPS5-4-3 (ABL74940.1)	47.33 / 29.24	zbmVI NRPS5-4 (ACG60776.1)	44.54 / 27.4
ORF116	580	blmV NRPS3CT (AAG02360.1)	42.7 / 27.03	TlmV NRPS3CT (ABL74945.1)	42 / 26.91	zbmV NRPS3CT (ACG60775.1)	43.61 / 28.8
ORF119	253	**thioesterase (AAG02346.1)**	**41.06 / 26.83**	-	-	**Zbm-Orf(-1) putative thioesterase (ACG60736.1)**	**47.09 / 28.7**

Table S4. C domain motifs of pAMDE1 in comparison to the BLM, TLM and ZBM motifs. Amino acids that differ from the classical C domain motif are shown in red. The numbers at the top are residue identifiers as described in SBSPKS server.

		conserved motif						
**DOMAIN**		**H**	**H**	**Xa**	**X**	**X**	**D**	**G**
BlmIV	(NRPS-2)	H	H	A	V	T	D	G
TlmIV	(NRPS-2)	H	H	I	A	I	D	G
ZbmIV	(NRPS-2)	H	H	A	V	T	D	G
**pAMDE1-108-C1**		**H**	**H**	**L**	**I**	**S**	**D**	**A**
BlmV	(NRPS-3,C0)	H	H	L	V	L	D	G
TlmV	(NRPS-3,C0)	H	H	L	I	L	D	G
ZbmV	(NRPS-3,C0)	H	H	L	I	L	D	G
**pAMDE1-116-C1**		**H**	**H**	**L**	**I**	**L**	**D**	**G**
BlmVI	(NRPS-3)	S	S	F	A	L	D	G
TlmVI	(NRPS-3)	S	S	F	G	L	D	G
ZbmVI	(NRPS-3)	S	S	F	G	L	D	G
**pAMDE1-115-C2**		**-**	**E**	**V**	**S**	**M**	**D**	**G**
BlmVI	(NRPS-4)	H	H	L	V	A	D	F
TlmVI	(NRPS-4)	H	H	L	L	A	D	F
ZbmVI	(NRPS-4)	H	H	L	V	A	D	Y
**pAMDE1-115-C1**		**H**	**H**	**A**	**I**	**C**	**D**	**F**
BlmVII	(NRPS-6)	H	H	I	A	S	D	G
TlmVII	(NRPS-6)	H	H	I	A	S	D	G
ZbmVII	(NRPS-6)	H	H	I	A	G	D	G
**pAMDE1-103-C1**		**H**	**H**	**I**	**C**	**S**	**D**	**G**
BlmIX	(NRPS-7)	H	H	I	V	F	D	G
TlmIX	(NRPS-7)	H	H	I	V	F	D	G
ZbmIX	(NRPS-7)	H	H	I	V	F	D	G
**pAMDE1-101-C1**		**H**	**H**	**L**	**I**	**I**	**D**	**E**
BlmX	(NRPS-8)	H	H	E	I	V	D	G
TlmX	(NRPS-8)	H	H	E	I	V	D	G
ZbmX	(NRPS-8)	H	H	E	I	V	D	G
**pAMDE1-100-C2**		**H**	**H**	**E**	**I**	**I**	**D**	**G**
BlmX	(NRPS-9)	H	A	L	V	A	D	R
TlmX	(NRPS-9)	H	A	L	V	G	D	R
ZbmX	(NRPS-9)	S	V	L	A	A	D	R
**pAMDE1-100-C1**		**P**	**A**	**M**	**F**	**G**	**D**	**K**

		cyclization conserved motif							
**DOMAIN**		**D**	**Xa**	**X**	**X**	**X**	**D**	**X**	**X**
BlmIV	(NRPS-0)	D	L	L	I	A	D	A	H
TlmIV	(NRPS-0)	D	L	L	I	A	D	A	H
ZbmIV	(NRPS-0)	D	L	L	I	A	D	A	H
**pAMDE1-108-C3**		**D**	**L**	**I**	**V**	**A**	**D**	**T**	**-**
BlmIV	(NRPS-1)	D	A	L	I	C	D	A	H
TlmIV	(NRPS-1)	D	A	L	I	C	D	A	Y
ZbmIV	(NRPS-1)	D	S	L	V	C	D	A	H
**pAMDE1-108-C2**		**Q**	**M**	**I**	**M**	**I**	**D**	**L**	**-**

Table S5. A domain motifs and amino acid specificity of pAMDE1 in comparison to that of BLM, TLM and ZBM. Amino acids that differ from the BLM, TLM and ZBM domain motifs are shown in red. The numbers at the top are residue identifiers as described in NRPSpredictor2 and SBSPKS server.

DOMAIN	235	236	239	278	299	301	322	330	331	517	SUBSTRATE
L-Cys(2)	D	L	Y	N	L	S	L	I	W	K	
BlmIV (NRPS-1)	D	L	Y	N	L	S	L	I	W	K	L-Cys(2)
TlmIV (NRPS-1)	D	L	Y	N	M	S	L	I	W	K	L-Cys(2)
ZbmIV (NRPS-1)	D	L	Y	N	L	S	L	I	W	K	L-Cys(2)
**pAMDE1-108-A2**	**D**	**L**	**Y**	**N**	**L**	**S**	**L**	**I**	**W**	**-**	**L-Cys(2)**
β-Ala	V	D	Xb	V	I	S	Xb	G	D	K	
BlmIV (NRPS-2)	V	D	W	V	I	S	L	A	D	K	β-Ala
TlmIV (NRPS-2)	V	D	W	V	V	S	L	A	D	K	β-Ala
ZbmIV (NRPS-2)	V	D	A	L	V	S	L	A	D	K	β-Ala
**pAMDE1-108-A1**	**V**	**D**	**A**	**T**	**V**	**S**	**I**	**A**	**D**	**K**	**β-Ala**
L-Asn	D	L	T	K	L	G	E	V	G	K	
BlmVI (NRPS-3)	D	L	T	K	V	G	E	V	G	K	L-Asn
TlmVI (NRPS-3)	D	L	T	K	V	G	E	V	G	K	L-Asn
ZbmVI (NRPS-3)	D	L	T	K	V	G	E	V	G	K	L-Asn
**pAMDE1-115-A3**	**D**	**M**	**T**	**K**	**L**	**G**	**E**	**V**	**G**	**K**	**L-Asn**
L-Asn	D	L	T	K	L	G	E	V	G	K	
BlmX (NRPS-9)	D	L	T	K	V	G	E	V	G	K	L-Asn
TlmX (NRPS-9)	D	L	T	K	V	G	E	V	G	K	L-Asn
ZbmX (NRPS-9)	D	F	T	K	V	G	E	V	G	K	L-Asn
**pAMDE1-100-A1**	**D**	**L**	**T**	**K**	**V**	**G**	**E**	**V**	**G**	**K**	**L-Asn**
L-Ser	D	V	W	H	L	S	L	I	D	K	
BlmVI (NRPS-4)	D	V	W	H	V	S	L	V	D	K	L-Ser
TlmVI (NRPS-4)	D	V	W	H	V	S	L	V	D	K	L-Ser
ZbmVI (NRPS-4)	D	V	W	H	L	S	L	I	D	K	L-Ser
**pAMDE1-115-A2**	**D**	**V**	**W**	**H**	**I**	**S**	**L**	**I**	**D**	**K**	**L-Ser**
L-Thr	D	F	W	N	I	G	M	V	H	K	
BlmVII (NRPS-6)	D	F	W	S	V	G	M	I	H	K	L-Thr
TlmVII (NRPS-6)	D	F	W	G	V	G	M	V	H	K	L-Thr
**pAMDE1-105-A1**	**D**	**A**	**W**	**Q**	**V**	**G**	**V**	**I**	**H**	**K**	**Gln**
L-Ala	D	L	F	N	N	A	L	T	Y	K	L-Ala
BlmIX (NRPS-7)	D	L	F	N	N	A	L	T	Y	K	L-Ala
TlmIX (NRPS-7)	D	L	F	N	N	A	L	T	Y	K	L-Ala
**pAMDE1-101-A1**	**D**	**V**	**F**	**T**	**Y**	**A**	**L**	**V**	**Y**	**K**	**Phe**
L-His	D	S	Xb	L	Xb	A	E	V	Xb	K	
BlmX (NRPS-8)	D	S	A	L	I	A	E	V	W	K	L-His
TlmX (NRPS-8)	D	S	A	L	V	A	E	V	W	K	L-His
ZbmX (NRPS-8)	D	S	V	L	T	A	E	V	W	K	L-His
**pAMDE1-100-A2**	**D**	**S**	**A**	**L**	**I**	**A**	**E**	**V**	**W**	**K**	**L-His**

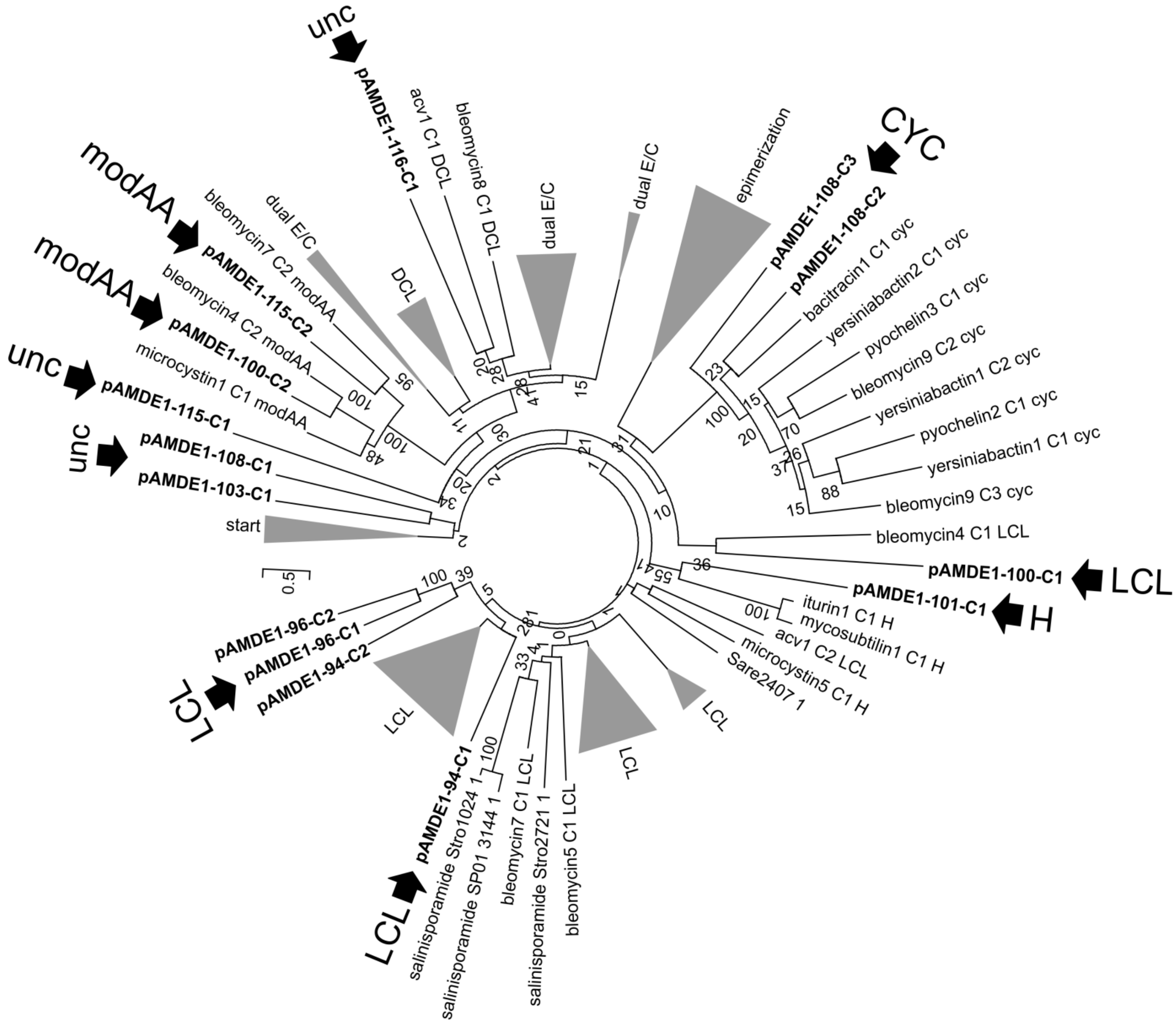

Figure S1. Phylogenetic tree of C domains. All known categories of C domains are shown. C domains of pAMDE1 are highlighted in bold. LCL: catalyzes peptide bond formation between 2 L-amino acids, DCL: catalyzes peptide bond formation between a D- and L- amino acid, CYC: heterocyclization domain, epimerization: flips chirality of last amino acid, dual E/C: catalyze both epimerization and condensation, modAA: modify the incorporated amino acids, H: hybrid, UNC: unclassified. Bootstrap values are shown on the branches.

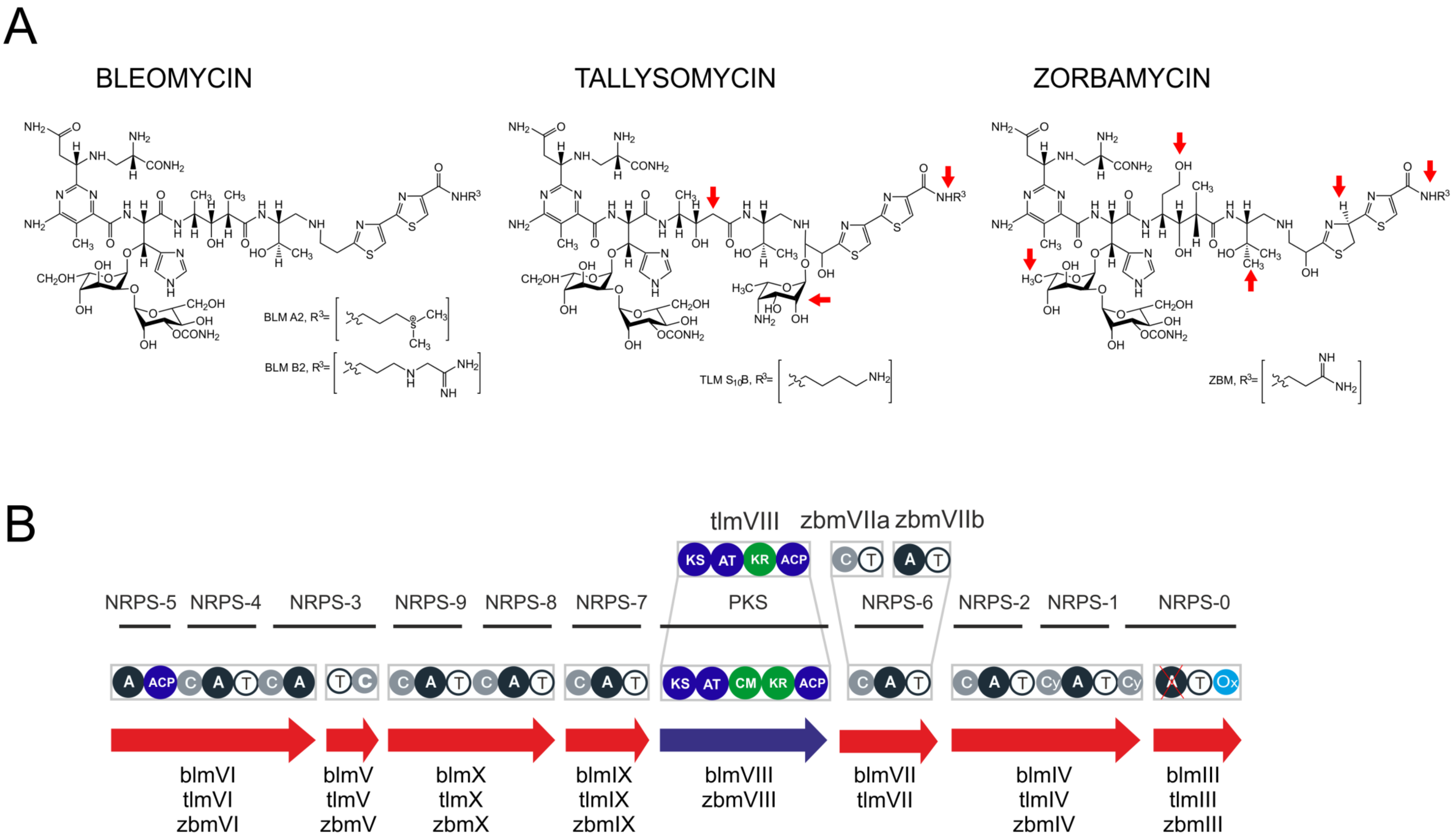

Figure S2. Bleomycin family of antitumor antibiotics. A) Structure of BLM, TLM and ZBM. Structural differences are highlighted with red arrows. B) Gene cluster representation and protein domains of bleomycin (blm), tallysomycin (tlm) and zorbamycin (zbm). The protein domains are shown inside the boxes. The modules (NRPS-0 to NRPS-9) described for the bleomycin compounds are represented by the black lines above the domains. The NRPS genes are represented by red arrows, and the PKS gene is represented by a blue arrow.

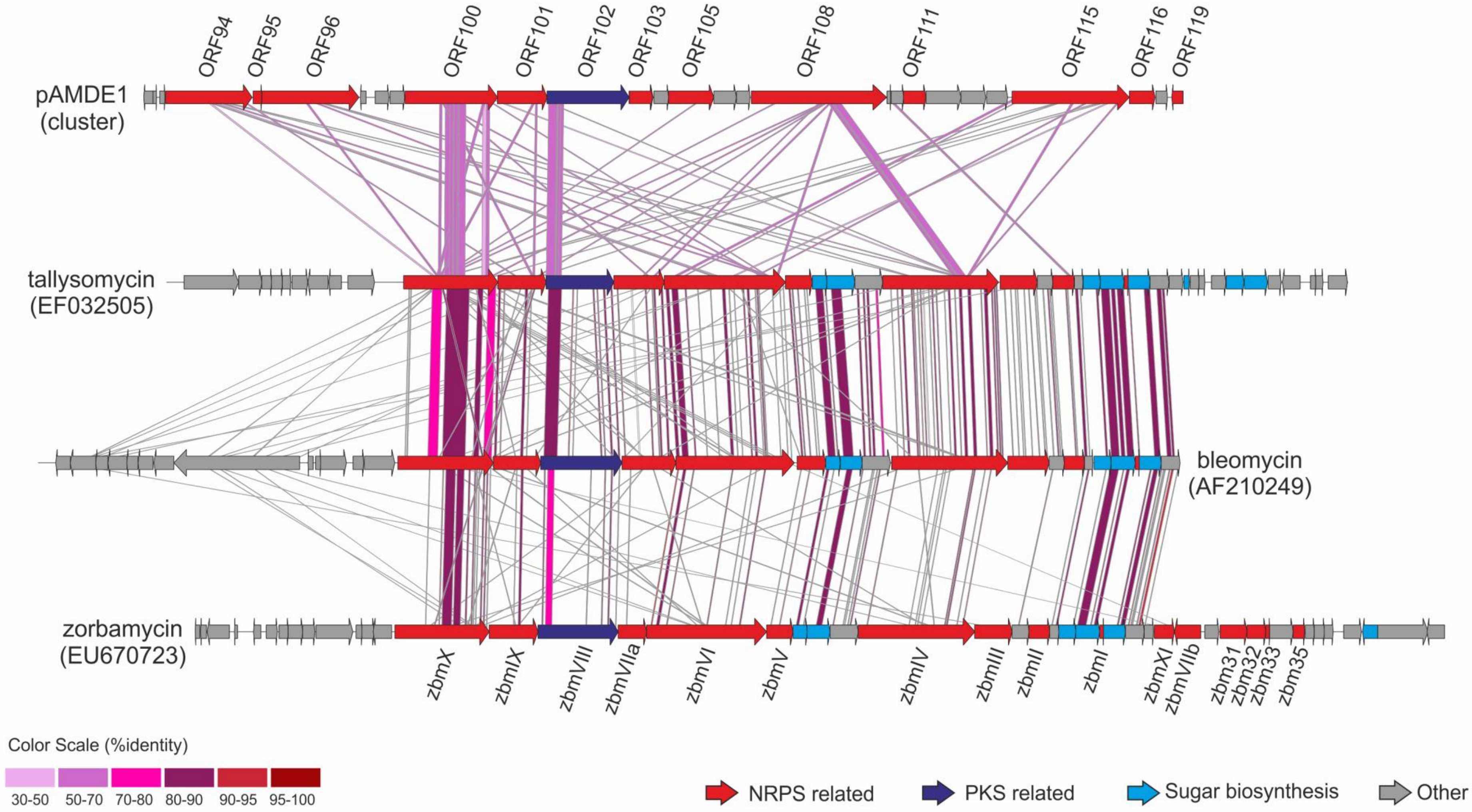

Figure S3. Cluster comparison of pAMDE1 to BLM, TLM and ZBM. All genes are colored according to the inferred function. Although all-versus-all comparisons (using BLASTN) were made, only selected pairwise comparisons are shown for clarity. The level of similarity between different contigs is indicated in the legend on the left. The name of the genes of pAMDE1 (represented by ORFs) and zorbamycin are shown.

```
Conservation:                                                                                                           5 337 4673523  3433
1MLA_chainA       1 -----------------------------------------------------------------------------------------------------QFAFVFPGQGSQTVGMLAD   19
3QAT_chainA       1 ----------------------------------------------------------------------------------------------------MGAAFTFPGQGSQLIGMGKV   20
Bleomycin_AT      1 ------PPAAP-RADRTAGVPMPLVVSARTREALAEAVRDLAAWSAPEPGTRLADLAATLAGR-RAFPYRAAVVCHDLPEAARLLGGARGE----TALPGREAVFLFPGQGTLPPDTGRG  108
Tallysomycin_AT   1 LEQ---PPAPP-LRAKRRAAQPLVLSARTGEALAEAARDLAERLRRDPGVDLADVAATLASR-RAFPHRKTVVCRDVSSAISALDLPNAPAR--VADAERPVVFLFPGQGAAYPGMARE  113
Zorbamycin_AT     1 LQE---SPAPARPRPE-PGGEAPLLLSGRDQDTLRRTADRLRRHLIEAPGTSLADIARTLSGK-RAFPHRRVVLAPDTATAVRALEAAPE-----EPAVARRVVFTLPDPTALSRADLA-  109
2JFD_chainB       1 -------------------SMRLLRASGRTPEAVQKLLEQGLRHSQDLAF--LSMLNDIAAVPATAMPFRGYAVLGGEGPEVQQVPA-----------GERPLWFICSGMGTQWRGMGLS   88
Alteromonas_AT    1 LQSYSEQKAET-GMAK-KAWPTVFPLSAKDPQSLQQYCASLVDNISAGDAPELSDLAYSLANRKTAFKHRVLLYAEDYADLRRQLEMLTTRSLSHQPENVTQFMLCLSGLNIESTNTVLN  118
Consensus_aa:       ....................................................................................................p.hhFhhsGbssb..sh..s
Consensus_ss:                             eeeee   hhhhhhhhhhhhhhhh      hhhhhhhhh         eeeee  hhhhhhhhh                eeeee       hhhhhh

Conservation:       3 3  3333  434  4333            2     42  2  4  23355273327633 5 3274    37223 956577337433792343 354353 254 22 3634
1MLA_chainA      20 MAASYPIVEETFAEASAALG---YD--LWALTQ---QGPAEELNKTWQTQPALLTASVALYRVWQQQGGK---APAMMAGHSLGEYSALVCAGVIDFADAVRLVEMRGKFMQEAVPEGTG  128
3QAT_chainA      21 LTEQFVAARMVFEEVDDALS---EK--LSDIIF---EGPADVLTLTANAQPALMAVSMAVIRVMEQLGLNVEKKVKFVAGHSLGEYSALCAAGTFSLTDTARLLRIRGNAMQAAVAVGEG  132
Bleomycin_AT    109 LYADVPAFRAHFDACAEGFA---PLGTDLHAAL------GAPADDTRAAQPALFAVEYALARTLMDWGV----RPAAMLGHSLGEYVAATLAGVLSLPDALTLVRARAEAQHTMPP---G  212
Tallysomycin_AT 114 LAREDVTFRAHLDECADLLA---GLGVELDAAL---DCPPGTVEATRLGQPALFAVEYALARALQDWGL----TPTAMLGHSLGEYVAATLAGVLSLPDALRLVSARGRVQAAMAP---G  220
Zorbamycin_AT   110 ALAAEPAVTRALDALIDRAA---RS--DVIVPR---PDDARASRPVTGAPATAFAVQYALVEALAGWGV----RPAAVSGAGTGELTAAVVAGMLTPVQALRLLTAATPGERAAAL----  213
2JFD_chainB      89 LMR-LDRFRDSILRSDEAVKPFGLK--VSQLLL---STDESTFDDIVHSFVSLTAIQIGLIDLLSCMGL----RPDGIVGHSLGEVACGYADGCLSQEEAVLAAYWRGQCIKEAHL-PPG  197
Alteromonas_AT  119 QFQADSALNRLLLRQLAAFG---LPSCGDIEKSLNSNNAKACLKG---YLIAYVAMTSAVVKYLTDADV----APGVFRGFGFSDFVAAYLAGAVTPAFLLQVIEEVADAIHG-------  221
Consensus_aa:       hh...shhp.hh......ht..........h.....pss..shp.h..sbsthhAhphAlhchh..hGl.....Pshh.G@thGEhsAhhhAGhlo.sphlpllph.tphbp.hh.....
Consensus_ss:       hhhh hhhhhhhhhhhhhh        hhhhh       hhhhhhhhhhhhhhhhhhhhhhhhhhh         eeeee  hhhhhhhhh     hhhhhhhhhhhhhhhhhh

Conservation:          9 3                33 33 34374 4234639   35  3 4 4   43     454236 7534634 43 3  39 23      47 233 234 4   432
1MLA_chainA     129 AMAAIIGLDDASIAKACEEAAEGQVVSPVNFNSPGQVVIAGHKEAVERAGAACKAAGAKRALPLPVSVPSHCALMKPAADKLAVELAKITF---NAPTVPVVNNVDVK--CETNG--DAI  241
3QAT_chainA     133 SMAALIGLDEKDVEEICEIVAEEGLCQIANDNGGGQIVISGEAKAVETAVEVASQKGAKRAVLLPVSAPFHSALMQPAANAMKNALLTVNK---TAPIVPLIANVSVI--PESDP--ERI  245
Bleomycin_AT    213 RMLAVP-LTPDDLRPLL-----PPEVEFSAFNAPGRCVVGGPPEPVAELRARLARRGV-PAAELATAHAFHSAAVEPLLDGFRGVLEGVRL---RPPRLRYVSSLTGD--WADAA--VTT  318
Tallysomycin_AT 221 RMLAVS-LPEDRLRPLL-----ASGLSVSAVNASDRCVVGGEPSAVADLAKALGGQGI-PTQMLPTAHAFHSTAVEPVLDRFREVLAEVEF---RAPSARYASNLTGN--WVRPEE-ACD  327
Zorbamycin_AT   214 ---ADPA---------------PSAPSLPCVGARSEGPVTGRHLLRPDFGAPAPGDTA-----EPVPHP------------------DPGP---GAPLLDVRLGLPGG--PASTP--ALR  285
2JFD_chainB     198 AMAA-VGLSWEECKQRC-----PPGVVPACHNSKDTVTISGPQAPVFEFVEQLRKE-VFAKEVRTGGMAFHSYFMEAIAPPLLQELKKVIREP-KPRSARWLSTSIPEAQWHSSLARTSS  309
Alteromonas_AT  222 -----------------------------------------DQSSTDQSIASLFDEIN------------FNRLMRE-SGRWQSLLDKLLIEGESSPAKLWWPSLAGE----PQH--HSL  281
Consensus_aa:       ...A..................ss.sp.sshst.spsslsG...sh.ph...h..p.h.....bshshs.@s.hhc...s.h...L.pl.....psP.h.hhsslss.....ss......
Consensus_ss:         eeee   hhhhhhhh        eeeeee     eeee  hhhhhhhhhhhhh    eeee         hhhhhhhhhhhhhhh           eeeee           h  hhh

Conservation:       32444 43       434      423 4 74453 29444365  33                         3 3 3
1MLA_chainA     242 RDALVRQLYNPVQWTKSVEYMAAQGVEHLYEVGPGKVLTGLTKRIVDTL------TASALN------EPSAMAAAL-------------------------------  305
3QAT_chainA     246 VSLLVQQVTGRVRWRETIEWISANGVNTLFEIGSGKVLTGLARRINKDI------KALTVG------TAEEIEAALRVLGV--------------------------  314
Bleomycin_AT    319 PAYWLAHLRRPVRFADGLRRCLDLGPVALVETGPRAGLTGLARRAAGPG------EPPYTV--RCLAAPDEAASLTHAVAVLWRSGCAVDWTAFHRPGRPRRTTVP-  416
Tallysomycin_AT 328 PDYWLAHMRQPVRFGDGLARCLELGPVSLVEVGPSAGLTGLVRRHPLASGDHRAVRTIPGA--RDSGEKSEVDSVVTAVAELWAEGCAVRWDAFHRQQEPRRVAVP-  431
Zorbamycin_AT   286 RTAW---------------------------------------SPPGPT----------------------GKGPVPVLAALWEAGVALKLDTFHRDRGARRATVPG  331
2JFD_chainB     310 AEYNVNNLVSPVLFQEALWHVP--EHAVVLEIAPHALLQAVLKRGLKPSC-----TIIPLMKKDHRDNLEFFLAGIGR---LHLSGIDANPNALFP-----PVEFP-  400
Alteromonas_AT  282 ADLLQTHIHK-----KAILTLCRAQSEHFINVCCS-QLLEPARQHDGTT----------------------NEILVTLVSRLWESGVELNWETLLPQYTAHHVRLP-  359
Consensus_aa:       .shh..pl.p.....ctl..h....s.hhhplts...Lh..h+p.s.s..........................t.l.h............................
Consensus_ss:       hhhhhhhh     hhhhhhhhhh    eeee    hhhhhhhhhh            ee           hhhhhhhhh  hhh
```

Figure S4. Structural alignment. Acyltransferase domains from four sequences of the bleomycin family and three known protein structures (PDB codes: 1MLA, 3QAT, 2JFD) are shown in the alignment. The alignment was created using the PROMALS3D web server. The first line in each block shows conservation indices for positions with a conservation index above 2. The last two lines show consensus amino acid sequence (Consensus_aa) and consensus predicted secondary structures (Consensus_ss). Consensus amino acid symbols are: conserved amino acids are in bold and uppercase letters; aliphatic: l; aromatic: @; hydrophobic: h; alcohol: o; polar residues: p; tiny: t; small: s; bulky residues: b; positively charged: +; negatively charged: -; charged: c. Known active site residues in the protein structure of 1MLA are indicated by a orange star symbol on top.
